# Human identification through forensic skeletal analysis: three case reviews

**DOI:** 10.1093/fsr/owae053

**Published:** 2024-08-29

**Authors:** Joe Adserias-Garriga, Shelby Feirstein, Dakota Bell, Hannah Skropits, Dennis C Dirkmaat

**Affiliations:** Department of Applied Forensic Sciences, Mercyhurst University, PA, USA; Department of Applied Forensic Sciences, Mercyhurst University, PA, USA; Department of Applied Forensic Sciences, Mercyhurst University, PA, USA; Department of Applied Forensic Sciences, Mercyhurst University, PA, USA; Department of Applied Forensic Sciences, Mercyhurst University, PA, USA

**Keywords:** forensic sciences, positive identification, radiography, computer tomography, surgical device, skeletal variation

## Abstract

Establishing a positive identification of human remains found in a forensic setting is often accomplished through DNA, fingerprints, or odontology. However, when these primary identifiers cannot be applied, practitioners can rely on combining points of concordance derived from other identification modalities such as antemortem trauma, pathology, or unique skeletal morphologies to build up a case for identification. In order to conduct these comparisons, forensic anthropologists must be well trained and experienced in human skeletal variation and antemortem trauma to properly evaluate a particular skeletal trait and understand its value with respect to personal identification. In addition to macroscopic analysis of skeletal features and standard radiographic images, recent forensic anthropological efforts of establishing personal identity from the skeleton have employed high-quality clinical imaging technologies. This article presents three forensic anthropological cases in which positive identification was established on the basis of multiple antemortem to postmortem comparison modalities that included skeletal variation, antemortem fracture morphologies, trabecular patterns, dental traits, and implanted surgical devices. These cases use a variety of imaging techniques, such as cranial radiographic images, dental radiographs, computed tomography, photography, and 3D surface scans of the skeletal remains, to achieve personal identification.

**Key points:**

- The identification of the remains becomes a top priority in forensic investigations.
- Dental treatment, implanted surgical devices, anatomical variation, and healed skeletal trauma can provide useful features for the antemortem *vs.* postmortem records comparison.
- This article discusses three cases in which multiple forms of antemortem and postmortem imaging were used to compare skeletal areas of interest.
- All cases were carried out by the Mercyhurst University Forensic Anthropology Team.

## Introduction

When human remains are found, their identification is one of the most critical steps in the forensic investigation. In fact, most cold cases involve unknown remains. According to the National Missing and Unidentified Persons System (NamUs), as of March 2024, there are over 24 000 missing persons and 14 600 unidentified persons reported throughout the USA [1].

In cases where the identity of the remains is unknown, especially cases involving fragmentary, burnt, severely decomposed, or skeletonized remains, forensic anthropologists and forensic odontologists are uniquely qualified to process and analyze the remains, and compare the postmortem to antemortem records as a means of identification, particularly when DNA analysis is not an accessible resource.

The International Criminal Police Organization (INTERPOL) Guide for Disaster Victim Identification (DVI), first published in 1984 and updated most recently in 2023, establishes fingerprinting, forensic odontology, and DNA as primary identifiers. Unique serial numbers from medical implants are also reliable primary identifiers. Secondary identifiers include any feature not categorized as a primary identifier that characterizes the individual, such as personal description and medical findings.

According to INTERPOL, the application of a single primary identifier (fingerprints, dental, or DNA analysis) or a combination of secondary identifiers are sufficient for establishing a positive identification or exclusion [2].

However, primary identifiers are not always available for skeletonized, fragmented, decomposed, or otherwise compromised remains [2, 3]. Nonetheless, a positive identification may still be possible through the analysis of antemortem skeletal trauma patterns and implanted surgical devices, as well as the combination of unique skeletal variations and anomalies within bones and teeth. Furthermore, new technologies and developments in the field of clinical imaging (eg, magnetic resonance imaging (MRI), computerized tomography (CT) scans) have provided another source of high-quality information useful for comparisons when managed appropriately.

Radiographic images are one of the most common sources of antemortem imaging. Radiographs create two dimensional images of the internal and external structures of the skeletal elements of interest. However, surrounding matrices may lead to the distortion of the image, particularly if the objects carry similar, or greater, densities than the surrounding skeletal tissue [4]. In recent years there has been a general trend toward the use of CT because of its ability to render greater detail of skeletal features compared to conventional radiography, as well as the possibility for composing 3D reconstructions of the skeletal elements included in the CT scan.

Anatomical structures or morphologies noted in radiographs and CT scans can be analyzed in conjunction with one another to work towards personal identification, in the form of a single unique identifier or multiple consistent features [5]. Still, the availability of CT scanners is not quite as ubiquitous as regular radiographs [6,7].

Comparative analysis of different imaging modalities relies on the consistencies and inconsistencies between antemortem and postmortem records in terms of overall bone morphology, patterns of trabeculae, unique features of the skeleton, and placement, orientation, and morphology of surgical devices.

Additionally, forensic odontologists interpret dental radiographs for comparison. Cranial and dental images are of special interest due to the wide range of structural variation within the dental arches and other cranial structures. Dental treatment, crown and root morphology, and the characteristics of surrounding anatomical structures present valuable information for the identification process. More specifically, the teeth that are present and absent, dental restorations, or the lack thereof, and tooth position in the dental arches can aid in positive identification [6].

While there is no standard for the number of points of concordance that permit a personal identification, it has been suggested that the postmortem to antemortem comparison of unique morphological features and locations of healed trauma should match in sufficient detail and lack irreconcilable inconsistencies [5,8]. Antemortem and postmortem images should also match on two levels of anatomical complexity: external shape and internal architecture [6, 9]. The greater the number of skeletal features in agreement between the antemortem and the postmortem images, and the more individualistic (that is, the less frequent in the population) those identifiers are, the greater the chance of rendering a positive identification.

Evidence of osteologically implanted surgical devices is sufficient for obtaining a positive identification in many cases. Orthopedic devices can carry manufacturer-specific information in the form of numbers etched onto the device that may be linked to a particular surgical office or an individual patient [6]. The Safe Medical Devices Act of 1990 (SMDA), administered through the Center for Devices and Radiological Health (CDRH) of Food and Drug Administration (FDA), requires the tracking of certain medical devices whose failure is likely to cause adverse health effects, is life sustaining, or is permanently implantable [10, 11]. Less than a decade later, the FDA Modernization Act of 1997 (FDAMA) allowed for the FDA to track devices to the patient level unless patients otherwise refuse the release of their medical records. And even though the identification numbering in surgical devices was originally created for clinical purposes, forensic professionals benefit from this information in the identification process. However, not all surgically implanted devices present this information on them; therefore, the identification will rely on radiological comparison.

This article will discuss three forensic cases in which positive identification was indicated through skeletal means, as well as a combination of identification modalities, including frontal sinus morphology, surgical devices, dental anatomy and restorations, callus formation, and overall skeletal variation for rendering positive identification through forensic anthropological analyses using and combining different imaging techniques.

## Case #1

Partially burned human remains were discovered by medicolegal personnel in an outdoor brick fire pit in the Northeast region of the USA. The remains exhibited significant thermal alteration consisting of charred and calcined skeletal elements, except for the distal portion of the lower limbs which were situated outside of the fire pit still fleshed and unburnt. The remains within the firepit showed a normal pattern of burning according to Schmidt and Symes [12]. According to the state of the unburned tissue on the lower legs and the information provided by the law enforcement investigation, the postmortem interval (PMI) was estimated to be between 24 and 48 h.

### Postmortem analysis

Burned remains are highly fragile and often fragmentary. Their processing requires expertise, time, and extreme care when handled during recovery, transportation, and lab analysis. In cases where the cranium and mandible are still articulated, with significantly charred tissue present, the tongue is frequently attached to the dentition in the lingual aspect, as it was in this case. In these circumstances, the best approach to radiographing the dental arches is *via* the submandibular approach, which requires accessing the teeth through the inferior and lingual aspect of the mandible.

**Figure 1:**
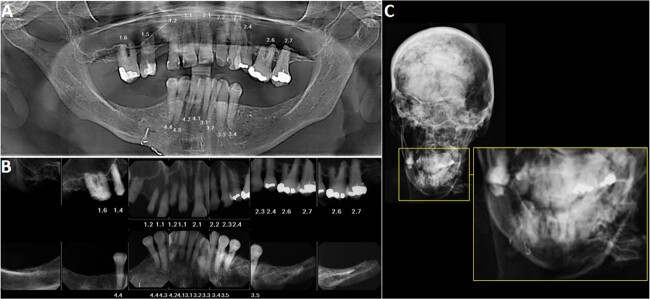
Radiographic images used for comparison in Case #1. (A) Antemortem panoramic radiograph, postmortem periapical radiographs. (B) Postmortem skull radiograph. (C) Detail of the skull radiograph showing the surgical wires (FDI/ISO dental numbering system).

**Figure 2:**
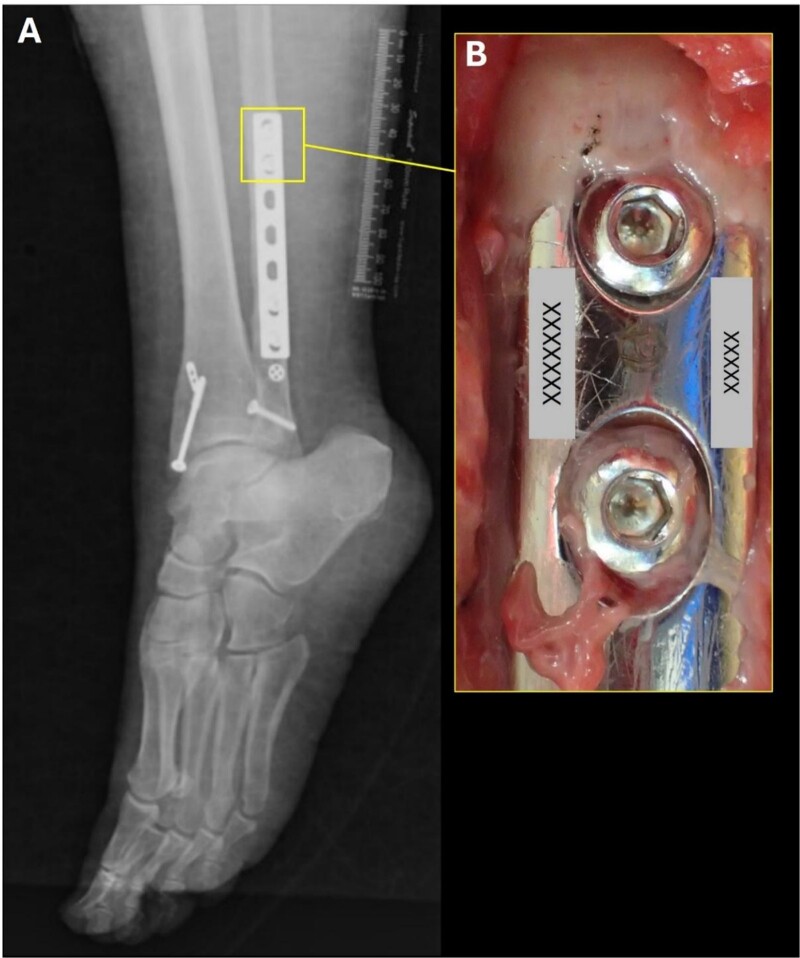
Postmortem radiographs of left distal lower limb, where the surgical plate fixed with screws and surgical ligament anchors can be observed (A). Detail photograph of the superior part of the surgical plate that showed the identification numbers indicated with “X” (B).

The dental radiographs in this case show that the dental crowns of teeth #1.6, 1.5, 1.2 and 1.1 (International Dental Federation/International Organization for Standardization (FDI/ISO) dental numbering system) (#3, 5, 7 and 8 in universal dental numbering system) were damaged by the fire exposure. However, teeth 2.4, 2.6 and 2.7 (FDI/ISO dental numbering system) (#12, 14 and 15 in universal dental numbering system) presented well-preserved restorations (Figure 1A). Additionally, two surgical wires were noted in the radiographic image of the mandibular body, indicating a healed fracture on the right side (Figure 1C). A surgical plate and screws were also noted on the distal-lateral aspect of the left fibula, containing serial numbering and the logo of the manufacturer (Figure 2).

Skeletal biological profile analyses were limited due to the significant thermal alteration, but the findings were consistent with an adult female.

### Antemortem data

Law enforcement had a tentative identification of the victim, so an antemortem panoramic dental radiograph was provided for comparison, taken 2 weeks before the remains were found. The antemortem radiograph showed teeth #1.8, 1.7. 1.4, 2.8, 3.8–3.5, 4.5, 4.8 (FDI/ISO dental numbering system) (#1, 2, 6, 16–20 and 29–32 in universal dental numbering system) were missing and dental restorations were present in teeth #1.1, 1.2, 1.5, 1.6, 2.4, 2.6 and 2.7 (FDI/ISO dental numbering system) (#3, 5, 7, 8, 12, 14 and 16 in universal dental numbering system) (Figure 1B). Additionally, surgical wires were noted on the right mandibular body. Hospital records were provided later, indicating the presence of an implanted surgical device in the distal left fibula and the associated identification numbers for the device.

### Comparison

Positive identification was achieved by comparing the antemortem panoramic and postmortem periapical radiographs and the postmortem pre-processing skull radiograph. Dental and cranial radiographs showed that the dental and maxillofacial anatomy, along with the location of dental restorations, morphology, and restorative material were consistent. The location of the surgical tie wires also matched. Furthermore, the identification numbers on the surgical plate in the distal left fibula corresponded with the identification numbers recorded in the hospital records. Antemortem radiographic images were not available for the left lower limb.

## Case #2

Human skeletal remains, with minimal soft tissue, were discovered by a construction crew at the bottom of a steep slope, near a small creek in a deciduous forest in the Eastern United States. According to the condition of the remains, which showed minimal dissecated soft tissue associated with mostly disarticulated remains, the PMI was estimated to be between 9 months and 2 years.

### Postmortem analysis

**Figure 3:**
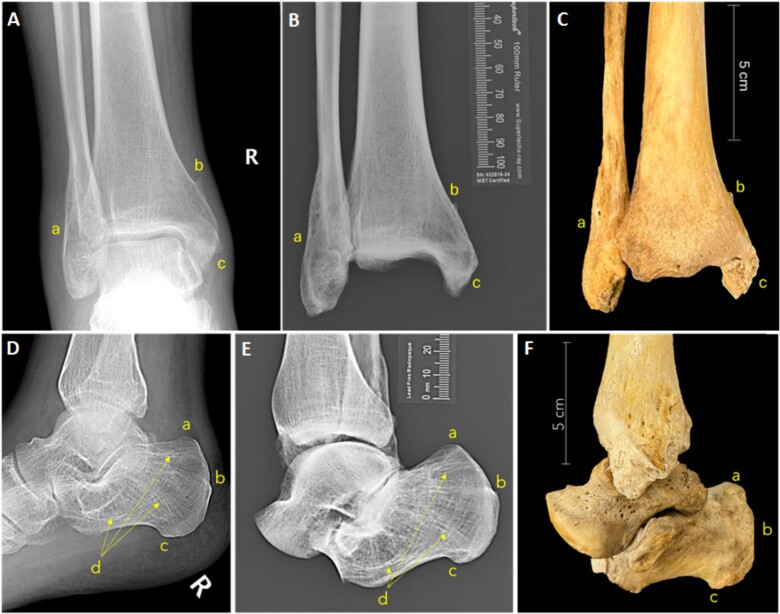
Antemortem radiographs (A, D), postmortem radiographs (B, E), and postmortem photographs (C, F) of the right distal lower limb including the tibia and fibula (A, B, C) and the calcaneus and talus (D, E, F). Fracture callus on the right distal fibula (a in A, B, and C), entheses on the right distal tibia (b and c in A, B, and C), and morphological traits of the calcaneus including entheses and trabecular pattern (a, b, c, d in D, E, and F).

**Figure 4:**
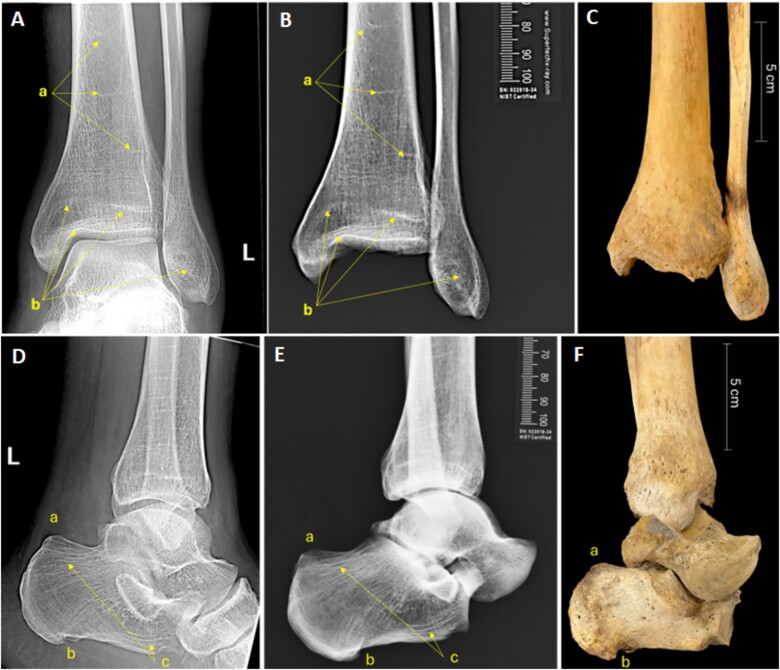
Antemortem radiographs (A, D), postmortem radiographs (B, E), and postmortem photographs (C, F) of the left distal lower limb including the tibia and fibula (A, B, C) and the calcaneus and talus (D, E, F). Harris lines in the distal tibia (a in A and B) as well as the overall trabecular pattern of the tibia and fibula (b in A and B). Overall trabecular pattern of the left calcaneus (a, b, c in D and E).

The biological profile analysis estimated a white male between 30 and 60 years of age at the time of death, who stood between 5′5″ (1.65 m) and 6′2″ (1.88 m) tall. The recovered remains presented signs of healed fractures on the left nasal bones and right distal fibula. Unique bone modifications were noted in the distal right tibia, right calcaneus and right talus in the form of periostitis associated with significant bone remodeling. Unique skeletal morphologies were noted in the distal left fibula, distal left tibia, and left calcaneus. No evidence of surgical intervention was present (Figures 3 and 4).

### Antemortem data

The antemortem records of the tentative victim provided by the local coroner’s office consisted of radiographs of the right and left ankle that were taken about one year prior to the recovery of the remains. The radiographs showed evidence of fracture calluses and bone modification in the form of entheses in the right distal tibia and calcaneus as well as Harris lines and unique trabeculae patterns in the left distal fibula, tibia, and calcaneus (Figures 3 and 4).

### Comparison

Antemortem ankle radiographs were compared with postmortem radiographs taken in the approximate same position. The location and overall morphology of the callus on the right distal fibula and the location and morphology of entheses on the right distal tibia and calcaneus were consistent. Furthermore, the appearance and location of Harris lines in the left distal tibia and fibula, as well as the overall trabecular patterns of the left tibia, fibula, and calcaneus were found to be consistent between the antemortem and postmortem radiographic images. Nasal fractures could not be evaluated for comparison since no antemortem imaging of the craniofacial area was available.

## Case #3

Human remains were discovered wrapped in a tarp inside a drainage ditch in the Northeast region of the USA. The remains were mostly skeletonized and disarticulated with minimal amounts of desiccated tissue and associated adipocere. Considering the condition of the remains and the scene information, the PMI was estimated to be less than 2 years.

### Postmortem analysis

The skeletal profile of the remains was estimated to correspond to a 30- to 50-year-old male who stood between 5′3″ (1.60 m) and 5′11″ (1.80 m) tall. No definitive result was obtained for the population affinity estimation of this individual. The remains presented healed fractures on the nasal bones and left zygomatic. Significant root resorption was noted in the maxillary and mandibular teeth in the area related to the maxillofacial trauma. Additionally, dental crowding was noted in the mandibular incisor and canine region along with a right accessory mental foramen.

### Antemortem data

Antemortem records of the suspected decedent were provided by the local coroner’s office, which included written medical records, cranial radiographs, and cervical CT scans taken approximately 8 years before the remains were recovered. The individual suffered a craniofacial trauma presenting multiple fractures to the facial structures that were evident in the radiographs and CT scans provided.

### Comparison

**Figure 5:**
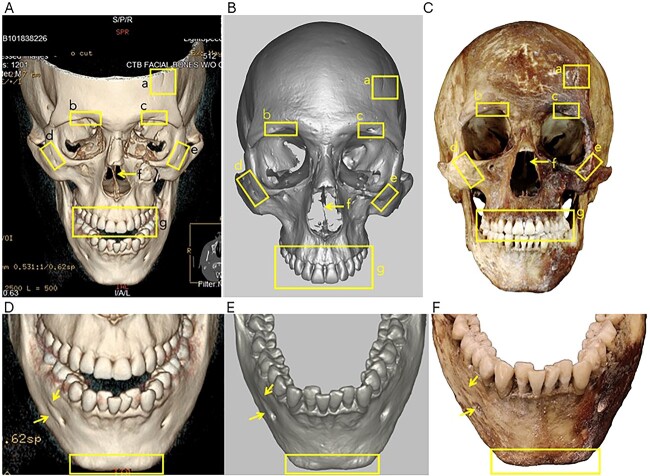
Antemortem CT scans reconstructions (A, D), postmortem 3D surface scans (B, E), and postmortem photographs (C, F) of the cranium and mandible. Highlighted skeletal morphology includes a frontal groove (a), supraorbital notch (b) and supraorbital foramen (c), two right zygomatic foramen (d) and one left zygomatic foramen (e), deviated septum (f), and overall maxillary dentition alignment and morphology (g). Mandible is shown in D, E, and F where yellow arrow highlights the presence of an accessory mental foramen and the yellow box shows overall mental morphology, additionally consistent mandibular dental crowding is noted in the antemortem and postmortem images.

**Figure 6:**
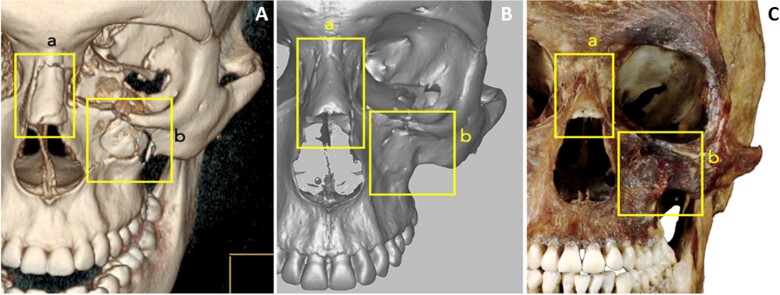
Antemortem CT scan reconstruction (A), postmortem 3D surface scan (B), and postmortem photography (C) of the left craniofacial structures. Highlighted in the yellow boxes are a nasal bone fracture (a) and a left maxillary fracture (b). The fractures appear unhealed in the antemortem CT scan (A) and healed in the postmortem images (B, C).

**Figure 7:**
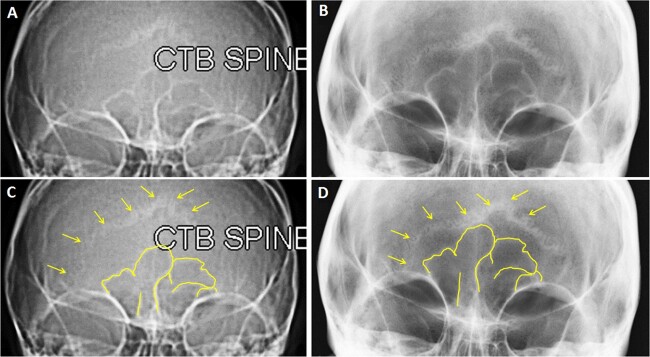
Antemortem radiographs (A, C) and postmortem radiographs (B, D) of the frontal sinus and lambdoidal suture. The frontal sinus is outlined in yellow and the lambdoidal suture is indicated by yellow arrows (C, D).

The antemortem and postmortem records showed strong consistencies in the location and overall morphology of the fractures, anatomical features, and the alignment of the teeth in the dental arches. The matching skeletal morphologies in the antemortem and postmortem records consisted of the presence and location of the left frontal groove (a in Figure 5), right supraorbital notch and left supraorbital foramen (b and c in Figure 5), two right zygomatic foramina and one left zygomatic foramen (d and c in Figure 5), as well as a right accessory mental foramen (Figure 5 double yellow arrows). Consistency was also observed in the nasal septum deviation (f in Figure 5), overall morphology and alignment of maxillary dentition (g in Figure 5), nasal bone fractures (a in Figure 6), left maxillary fracture (b in Figure 6), and dental morphology and alignment in the mandibular arch, especially in the anterior teeth showing dental crowding (Figure 5D, E, F). Finally, the overall morphology of the mental protuberance (Figure 5D, E, F), frontal sinus morphology (Figure 7), and lambdoidal suture pattern were consistent (Figure 7).

## Discussion

In cases involving fragmentary, burnt, severely decomposed, or skeletonized remains, forensic anthropologists and forensic odontologists are uniquely qualified to assist in the identification process. The different types of comparisons reviewed in this article include surgical implements, trauma fracture patterns, callus formation, trabeculae pattern, skeletal anomalies, as well as frontal sinus morphology and cranial suture patterns. When analyzing these identifiers, practitioners should consider the following:

Heat-altered remains are often structurally compromised and require extreme care when handling. Thermal alteration causes colour, dimensional, and structural changes in bone, with calcined bones being the most brittle [12, 13]. Caution should be taken when conducting analyses and comparative materials should be utilized to identify fragments with confidence.

Trauma can be assessed based on the characteristics and morphology of an injury. Antemortem refers to trauma experienced during life that shows signs of healing and remodeling. In living bone, healing occurs in a predictable set of stages: inflammatory, reparative, remodeling, and healing. Remodeling is the slowest phase of healing which attempts to arrange unorganized bone into organized cortical bone along lines of stress. However, the newly formed mature callus will never match the structure of the surrounding unfractured bone.

Surgical devices provide insight into antemortem injuries or disease. It is the practitioner’s responsibility to understand what pathologies, injuries, or other compounding trauma may lead to common surgical devices. Though some devices consist of multiple manufacturers’ parts, thusly complicating the individuation process, many have corporate identifiers that can narrow down the search for a former patient [10].

Anatomical variations can be observed in the bone using advanced technologies such as radiographs, CT scans, or MRI. Forensic practitioners ought to learn how each of these technologies function both as separate entities, and in conjunction with one another for rendering a positive identification. Different cranial and postcranial anatomical features have been used for forensic human identification including cranial sutures pattern, frontal, sphenoidal, nasal, and mastoid sinuses; hand and wrist, vertebrae, and leg and foot elements [14–16].

Frontal sinuses continue to be a well-recognized area of interest for personal identification. This structure is located superior to the cranial landmark nasion, in the supraorbital ridge region and presents significant variation ranging from minimal to large intricate labyrinthian matrices. This feature is so individualizing that identical twins will display unique expression despite their shared genetic makeup. The antemortem *vs.* postmortem comparison of this structure can be carried out on 2D and 3D images [6,14,15].

The cases included in this paper represent positive identifications achieved through the skeletal analysis of the remains, and they exemplify the essential proficiency forensic anthropologists must have both in combining 2D and 3D imaging techniques and in analyzing multiple skeletal features identifying consistencies and inconsistencies and assigning their proper value for the identification assessment. Forensic anthropologists and odontologists must have the expertise to distinguish highly individualistic traits from those that are more common. These two skills (recognizing unique anatomical features and effectively employing advanced imaging techniques) contribute to a robust identification process.

## Conclusions

Proper use of antemortem and postmortem data can assist in rendering positive identifications in complex cases. However, a forensic practitioner must know how to interpret the images, as well as when to employ each method of comparison to accomplish the identification. While fingerprints, odontology and DNA are considered the primary identifiers, other modalities of antemortem and postmortem comparison may render a positive identification. Certain skeletal traits have been established as highly individualistic such as dental treatment and morphology, frontal sinus morphology and structure, fracture callus morphologywand characteristics of implanted surgical devices.

However, there is a lack of knowledge on how frequent certain anatomical variants are in a population, which is key for establishing a hierarchy of different anatomical traits according to their frequency: the more frequent the trait is, the less individualistic it is. Therefore, the least frequent anatomical variants are the most valuable ones for identification purposes. Though data on the frequency of appearance of certain skeletal traits is lacking, combining different anatomical features equates to higher robustness during comparative analysis. Further research is needed on this topic to emphasize the objectivity of the identification process.
